# Insurance Churn and Diabetes Outcomes Among Patients With Low Income

**DOI:** 10.1001/jamahealthforum.2026.0034

**Published:** 2026-03-20

**Authors:** Nathalie Huguet, Dang Dinh, Annie Larson, Andrew Suchocki, Jun Hwang, Jennifer DeVoe, Miguel Marino

**Affiliations:** 1 Department of Family Medicine, Oregon Health & Science University, Portland; 2 Research Department, OCHIN Inc, Portland, Oregon; 3 Clackamas Health Centers, Oregon City, Oregon; 4 Statistical Editor, *JAMA Health Forum*

## Abstract

This case-control study assesses the association of losing insurance with diabetes outcomes among people with low income served by community health centers.

## Introduction

Evidence shows that people with low income who have diabetes are 25% more likely than those without to lose health insurance.^1^ Changes to Medicaid and health insurance exchange programs via the One Big Beautiful Bill Act will lead to unstable insurance access. This study assesses the association of losing insurance (“churning”) with diabetes outcomes among people served by community health centers (CHCs), 90% of whom have incomes at or near the federal poverty level.^2^

## Methods

This case-control study uses electronic health record (EHR) data from January 1, 2014, to December 31, 2019, from 354 CHCs in 20 states in OCHIN and Health Choice Network on patients with diabetes aged 19 to 64 years. Patients had an *ICD-9-CM* or *ICD-10-CM* code on the problem list and/or encounter diagnoses any time between January 1, 2012, and December 31, 2017. Patients must have had 3 or more ambulatory visits within a 3-year period and an interval of 12 months or more between the first and last visit. Postperiod included any visits after churning up to December 31, 2019 (eMethods in Supplement 1). Oregon Health & Science University’s institutional review board approved the study and waived informed consent because the data were deidentified. This study followed the STROBE reporting guideline.

Diabetes outcomes included uncontrolled glycosylated hemoglobin (HbA_1c_) levels (>9.0%), insulin prescriptions, prescriptions for diabetes medications categorized by regimen complexity (eg, required prior authorization or demonstrated nonresponse to prior medication), and acute diabetes-related complications, identified using *ICD-9-CM* or *ICD-10-CM* codes.^3^

Data were analyzed from October 2024 to December 2025. EHR-based health insurance data were collected at each visit for billing.^4^ *Churn* was defined as 2 or more consecutive uninsured visits, to capture sustained coverage loss. *Nonchurn* was defined as every visit insured (n = 27 939) or a single uninsured visit (between visits, n = 5648; last visit, n = 1130). To construct analogous preintervals or postintervals for the nonchurn group, we assigned a pseudo-churning date in any year with 2 or more visits to match the churn group definition. Propensity score matching was used to create comparable groups.

Pre-post changes in outcomes between propensity score–matched patients in the churn and nonchurn groups were compared using generalized estimating equations logistic regression, adjusting for covariates used in matching procedure to reduce residual confounding. Variables and modeling specifications are described in eMethods in Supplement 1. Standard errors were clustered at the patient level. We calculated average marginal effects from logistic models to allow for estimation of absolute difference in outcome prevalence from preperiod to postperiod between groups.

## Results

There were 39 144 patients (churn group: n = 5557; mean [SD] age, 48.2 [8.3] years; nonchurn group: n = 33 587; mean [SD] age, 48.6 [8.8] years). The churn group experienced significantly worse outcomes over follow-up, including higher prevalence of uncontrolled HbA_1c_ (pre-post difference between churn and nonchurn groups, 4.0% [95% CI, 2.3%-5.8%]), acute complications (difference, 3.0% [95% CI, 1.0%-5.0%]), and insulin prescriptions (difference, 2.2% [95% CI, 0.8%-3.6%) (Table).

**Table.** Propensity Score–Matched Adjusted Prevalence of Diabetes Outcomes Postchurn vs Prechurn Among Patients With Diabetes, 2014-2019^a^

Group	Adjusted prevalence (95% CI), %^b^			
	HbA_1c_ >9.0%^c^	≥1 Acute complication^d^	Diabetes medication (high complexity)^e^	Insulin prescription^f^
Churn				
Preperiod	33.5 (32.2 to 34.8)	12.9 (12.0 to 13.8)	13.3 (12.4 to 14.3)	34.0 (32.7 to 35.4)
Postperiod	34.0 (32.7 to 35.3)	27.3 (26.1 to 28.6)	22.0 (20.8 to 23.2)	42.4 (41.0 to 43.8)
Postperiod-preperiod difference	0.5 (−0.8 to 1.8)	14.4 (13.0 to 15.9)	8.7 (7.5 to 9.8)	8.4 (7.4 to 9.4)
Nonchurn				
Preperiod	33.3 (32.0 to 34.7)	12.7 (11.8 to 13.6)	12.9 (12.0 to 13.9)	32.8 (31.5 to 34.1)
Postperiod	29.8 (28.5 to 31.1)	24.2 (23.0 to 25.4)	21.1 (19.9 to 22.2)	39.0 (37.6 to 40.3)
Postperiod-preperiod difference	−3.5 (−4.7 to −2.3)	11.5 (10.1 to 12.9)	8.1 (7.1 to 9.2)	6.2 (5.2 to 7.1)
Postperiod-preperiod difference between churn and nonchurn groups	4.0 (2.3 to 5.8)^g^	3.0 (1.0 to 5.0)^g^	0.5 (−1.0 to 2.1)	2.2 (0.8 to 3.6)^g^

Abbreviation: HbA_1c_, glycosylated hemoglobin.

SI conversion factor: To convert HbA_1c_ to proportion of total hemoglobin, multiply by 0.01.

^a^ The preperiod determines baseline outcomes from the first insured visit (via Medicaid or private insurance) in the study period through the churning date (or pseudodate). Postchurning assessment among the churn and nonchurn groups occurred at a mean (SD) follow-up of 35.0 (4.4) months. The churn group was defined as having 2 or more consecutive uninsured visits. The nonchurn group included patients who had every visit insured or a single uninsured visit.

^b^ Adjusted prevalences are average marginal effects derived from a generalized estimating equations logistic model adjusted for sex, age, race and ethnicity, baseline insurance type, patient rural or urban location, multimorbidity, and number of visits in the preperiod.

^c^ Adjusted prevalence of patients with HbA_1c_ level higher than 9.0% relative to 9.0% or lower.

^d^ Adjusted prevalence of patients with 1 or acute diabetes complications relative to no acute diabetes complications.

^e^ Adjusted prevalence of patients with highest complexity diabetes medication regimen relative to medium or lowest complexity.

^f^ Adjusted prevalence of patients with an insulin prescription relative to none.

^g^ Statistically significant at α = 5%.

## Discussion

This case-control study found that insurance churn among CHC patients with diabetes was associated with poorer diabetes management and increased insulin use and acute diabetes complications. These results underscore the harm insurance (Medicaid or private) instability could have on CHC patients with diabetes. Loss of Medicaid funding could also strain CHC resources due to loss of revenue, impacting patient care.^5^ Supporting continuous insurance coverage for patients with low income who have diabetes will likely lower risk for costly preventable diabetes complications.

Limitations include restricting the sample to patients with 3 or more ambulatory visits, lack of information received outside this network, and potential misclassification of the churn group. Alternative model and churn specifications are possible^6^ but were limited by irregular outcome timing; however, sensitivity analyses in a smaller subset produced directionally consistent results, supporting the findings’ robustness.

## Notes

Supplement 1.
eMethods.

eReferences.

Supplement 2.
Data Sharing Statement
