# Supplementary material for: Insurance Churn and Diabetes Outcomes Among Patients With Low Income
Source: JAMA Health Forum. 2026 Mar 20;7(3):e260034. doi: 10.1001/jamahealthforum.2026.0034 (PMC13005156; doi:10.1001/jamahealthforum.2026.0034)
Supplement: Supplement 1. — eMethods. eReferences. [file jamahealthforum-e260034-s001.pdf]

Supplementary Online Content

Huguet N, Dinh D, Larson A, et al. Insurance churn and diabetes outcomes among patients with low income. *JAMA Health Forum.* 2026;7(3):e260034. doi:10.1001/jamahealthforum.2026.0034

eMethods.
eReferences.

This supplementary material has been provided by the authors to give readers additional information about their work.

© 2026 Huguet N et al. *JAMA Health Forum.*

eMethods.

This retrospective cohort study uses electronic health record (EHR) data from the Accelerating Data Value Across a National Community Health Center Network (ADVANCE) of community health centers (CHCs).[1] ADVANCE data are from OCHIN (not an acronym) and Health Choice Network (HCN). OCHIN offers fully hosted and tailored OCHIN Epic practice management and EHR solutions. Similarly, HCN consists of a group of CHCs on a single EHR system. The data from OCHIN and HCN are centralized and standardized in the ADVANCE data warehouse using the PCORnet common data model. The profile of patients in the ADVANCE data are comparable to national estimates of CHC[2] patient characteristics with regard to diabetes prevalence, poverty, payer mix, race and ethnicity, and age.[3-5]

We extracted data for 1,713,977 patients aged 19–64 seen in 354 clinics across 20 states (Medicaid expansion and non-expansion states) between 01/2014 and 12/2019. This study period is inclusive of the Affordable Care Act Medicaid expansion and exclusive of the COVID-19 pandemic and post-Public Health Emergency periods, which greatly impacted Medicaid enrollment. Patients who were pregnant, had Medicare coverage, or had another type of insurance (e.g., Tricare, grant program) during the study period were excluded as they have different health care needs and access options.

Patients with diabetes had an ICD-9-CM or ICD-10-CM code on the problem list and/or encounter diagnoses any time between 2012 and 2017 (97% with diabetes type 2). The years 2012 and 2013 were used to determine diabetes status in 2014. Patients must have had ≥3 ambulatory visits within a 3-year period during the study period (2014-2019), and ≥12-month interval between the first and last visit during the study period. Baseline period spanned 2014 to 2016 and post-period spanned 2014 to 2019, allowing all patients up to a 3-year post-period. The pre-period determines baseline outcomes from the first insured visit (via Medicaid or private coverage) in the study period through the churning date (defined below). The post-period spanned from the churning date until either 3 years had elapsed or until December 31, 2019, whichever came first (the mean pre- and post-period lengths of 12.8 and 34.5 months for churners and 12.1 and 35 months for non-churners with averages of 4 pre- and 10 post-period encounters for both churners and non-churners). Post-churning assessment among churners and nonchurners averaged a follow-up of 35 months. This longer post-period allowed patients to have ambulatory visits regardless of insurance status and for outcomes to occur over a longer period than the baseline to observe longterm impacts of loss of insurance. Our population of interest includes patients who utilize healthcare infrequently enough to necessitate the longer, single pre- (and single post-period) per patient in our cohort analysis. Although many patients had multiple observations during their pre-periods, requiring measurements across multiple, shorter pre-period windows reduced our sample size by more than half, disproportionately excluding patients with low care utilization (the population most vulnerable to churning and central to our research question).

Our primary independent variable distinguished patients who churned out of health insurance coverage versus those who did not. Those who churned ("churners," N=5,557) were defined as having ≥2 consecutive uninsured visits. Those who did not churn ("nonchurners" N=33,587) included patients who had every visit insured (27,939) or no more than one uninsured visit in the study period (5,648). Among this last group, the uninsured visit could have been in between insured visits (median number of months between visits <2 months), possibly due to delay in enrollment (n=4,518), or as their last visit (N=1,130). Data on health insurance status from the EHR are primarily based on information collected at each visit for billing purposes,[6] represent a reliable source of information on insurance status and services received at each visit, and have demonstrated excellent agreement with Medicaid data in CHCs.[7] We chose 2 consecutive uninsured visits to better capture the concept of "true" loss of coverage rather than short administrative gaps or delays in re-enrollment. Patients in CHCs can experience smaller period of uninsurance due to delay in re-enrollment. We wanted to exclude these instances. Many CHCs have eligibility specialists that can assist patients re-enroll into Medicaid which is the bulk of their work.[8]A definition based on a single uninsured visit would potentially misclassify many patients who were not meaningfully uninsured. Among 10,292 patients with at least one such instance, 75% regained insurance within six months, demonstrating that a single uninsured visit often reflects temporary administrative interruption rather than true churn. Additionally, 1,546 instances occurred as a patient's final visit, making follow-up impossible. Further, while a one-visit definition is often used in survey data research with 12-month recall windows, our visit-based EHR data allow us to distinguish transient gaps from more sustained coverage loss. For this reason, we required two consecutive uninsured visits.

We randomly assigned a pseudo-churning date to nonchurners to create analogous "pre" and "post" periods for both

© 2026 Huguet N et al. *JAMA Health Forum.*

groups. The churning date for churners is the first uninsured visit date of two consecutive uninsured visits. Since churners are defined as patients with at least two consecutive uninsured visits, we assigned a pseudo-churning date to nonchurners in any year in which they have at least two consecutive visits, thereby mimicking the temporal structure necessary for matching and leading to nonchurners having multiple potential pseudo-churning dates. For instance, a nonchurner with two consecutive visits in 2014 and then two consecutive visits in 2016, would have two possible matching pseudo-churning dates. We then used propensity score matching to create equivalent groups. Propensity scores were estimated using logistic regression, modeling the probability of being a churner based on baseline covariates listed below along with pre-period outcomes (defined below).[9] We performed 1:1 nearest-neighbor matching with replacement to pair churners with nonchurners who had similar propensity scores. This approach aimed to reduce confounding by balancing observed characteristics between churning groups before performing logistic regressions on the matched sample.

We examined four diabetes outcomes across the pre- and post- (pseudo) churning periods:
(1) Uncontrolled glycosylated hemoglobin levels assessed in accordance with the Centers for Medicare & Medicaid Services (CMS) quality metric to determine uncontrolled diabetes status (defined as HbA1c > 9.0)[10]
(2) Any prescription for insulin
(3) Any prescription for diabetes medications categorized by the complexity of the medication regimen (e.g., required prior authorization or demonstrated nonresponse to prior medication such as DPP4 inhibitors, metformin-DPP4, SGLT2)
(4) The occurrence of any acute diabetes-related complications (abnormal blood glucose, acute kidney failure, cardiac arrest, cardiac arrythmia, congestive heart failure, diabetic ulcer, glaucoma, hyperkalemia, hypertensive emergency, hypotension or shock, infections or closely related conditions, myocardial infarction, neuropathy, noncardiac, noncerebral artery complications, stroke, transient neurological deficit, or cerebral artery occlusion). Acute complications were identified using ICD-9-CM and ICD-10-CM code classifications, had to occur on or after the first diagnosis of diabetes, and were counted as distinct complications if the interval between diagnostic encounters was at least 10 days.[11]

Characteristics of patients include sex, age, race and ethnicity (self-reported), federal poverty level, and patient rural/urban residential classification. We assessed multimorbidity status (2+ conditions excluding diabetes diagnosis), baseline payor type (Medicaid or private), and the average number of ambulatory visits in the pre-period.

Statistical analysis

We first compared the characteristics of churners and nonchurners after propensity score matching to assess covariate balance. We then estimated the association between insurance churn and outcomes via GEE logistic regression models on the matched sample. Regression data were structured at the patient-period level, where a single pre- and single post-churn period were constructed and summarized per patient, so that the analytic dataset contained two entries per patient. This approach compared pre- and post-changes in outcomes among patients who experienced churn (churners) with those who did not (nonchurners). The regression models included indicators for time period (pre- vs. post-churn or pseudo-churn), churn status, and their interaction term (churn status x period) to capture the estimate. Models also adjusted for covariates used in the matching procedure to reduce residual confounding and improve statistical efficiency. These covariates are described above and include demographic and health-related characteristics such as sex, age, race and ethnicity, federal poverty level, rural or urban residence, multimorbidity status (defined as ≥2 chronic conditions excluding diabetes), pre-churn payer type (Medicaid vs. private insurance), and the average number of ambulatory visits during the pre-period. Our methodological approach described above can be specified in the following form:

$$\begin{aligned}\text{logit}(\text{P}(\text{outcome}) \\ &= \beta_0 + \beta_P(\text{postperiod}) + \beta_C(\text{churner}) + \beta_{PC}(\text{postperiod} * \text{churner}) + \beta_1(male) \\ &+ \beta_2(agecategory) + \beta_3(raceethnicity) + \beta_4(FPL\ category) + \beta_5(patient\ location) \\ &+ \beta_6(multimorbidity\ category) + \beta_7(private\ preperiod\ insurance) \\ &+ \beta_8(number\ of\ preperiod\ visits)\end{aligned}$$

Categorical variables are presented as single indicators above for convenience, but are actually included in the model as sets of covariates with dummy/binary indicators; categories are sex (female, Male), age (19-44, 45-64), race and ethnicity (non-Hispanic White, Hispanic, non-Hispanic Black, non-Hispanic Other, missing), baseline insured visit (Medicaid, private), federal poverty level (≤1385, >138%, missing), patient location (large rural, small

© 2026 Huguet N et al. *JAMA Health Forum.*

rural, suburban, urban), multimorbidity at baseline (mental conditions only, physical conditions only, mental and physical, none).

Standard errors were clustered at the patient level to account for repeated observations (using an independent working correlation structure, which is appropriate when each subject contributes only two time points and the correlation structure cannot be reliably estimated). For interpretability, we calculated and reported average marginal effects by group (churners vs. nonchurners) and time period (pre- vs. post-churn). Average marginal effects allowed for comparisons of adjusted prevalences on the additive scale, so that each result could be interpreted as a difference-in-differences (DID). Because our study design required only a single measurement in the pre-period per patient, we were unable to formally assess parallel trends in outcome trajectories prior to churn, however, sensitivity analyses in a smaller subset produced directionally consistent results (not shown), supporting the robustness of our findings. For marginal effects and odds ratios, we report 95% confidence intervals. Analyses were conducted using R (R Core Team, 2021) and Stata version 17.0 (StataCorp, 2021).

© 2026 Huguet N et al. *JAMA Health Forum.*

eReferences.

1. DeVoe JE, Gold R, Cottrell E, et al. The ADVANCE network: accelerating data value across a national community health center network. *J Am Med Inform Assoc*. Jul-Aug 2014;21(4):591-5. doi:10.1136/amiajnl-2014-002744
2. National Association of Community Health Centers. Community Health Center Chartbook 2024. Accessed May 20295, 2025. https://www.nachc.org/resource/community-health-center-chartbook/
3. Chamine I, Hwang J, Valenzuela S, et al. Acute and Chronic Diabetes-Related Complications Among Patients With Diabetes Receiving Care in Community Health Centers. *Diabetes Care*. Oct 1 2022;45(10):e141-e143. doi:10.2337/dc22-0420
4. Huguet N, Dinh D, Hwang J, et al. The Impact of the Affordable Care Act Medicaid Expansion on Acute Diabetes Complications Among Community Health Center Patients. *J Prim Care Community Health*. Jan-Dec 2023;14:21501319231171437. doi:10.1177/21501319231171437
5. Quinones AR, Hwang J, Huguet N, et al. Diabetes Complications Among Community-Based Health Center Patients with Varying Multimorbidity Patterns. *J Gen Intern Med*. May 2025;40(6):1350-1358. doi:10.1007/s11606-025-09457-y
6. Heintzman J, Marino M, Hoopes M, et al. Supporting health insurance expansion: do electronic health records have valid insurance verification and enrollment data? *J Am Med Inform Assoc*. Jul 2015;22(4):909-13. doi:10.1093/jamia/ocv033
7. Marino M, Angier H, Valenzuela S, et al. Medicaid coverage accuracy in electronic health records. *Prev Med Rep*. Sep 2018;11:297-304. doi:10.1016/j.pmedr.2018.07.009
8. Huguet N, Valenzuela S, Marino M, et al. Effectiveness of an insurance enrollment support tool on insurance rates and cancer prevention in community health centers: a quasi-experimental study. *BMC Health Serv Res*. Oct 30 2021;21(1):1186. doi:10.1186/s12913-021-07195-5
9. Stuart EA. Matching methods for causal inference: A review and a look forward. *Stat Sci*. Feb 1 2010;25(1):1-21. doi:10.1214/09-STS313
10. Centers for Medicare & Medicaid Services. Quality ID #1 (NQF 0059): Diabetes: Hemoglobin A1c (HbA1c) Poor Control (>9%). Accessed August 7, 2024. https://qpp.cms.gov/docs/QPP_quality_measure_specifications/CQM-Measures/2022_Measure_001_MIPSCQM.pdf
11. Wharam JF, Zhang F, Eggleston EM, Lu CY, Soumerai S, Ross-Degnan D. Diabetes Outpatient Care and Acute Complications Before and After High-Deductible Insurance Enrollment: A Natural Experiment for Translation in Diabetes (NEXT-D) Study. *JAMA Intern Med*. Mar 1 2017;177(3):358-368. doi:10.1001/jamainternmed.2016.8411

© 2026 Huguet N et al. *JAMA Health Forum.*
