# Supplementary material for: Insurance Churn and Diabetes Outcomes Among Patients With Low Income
Source: JAMA Health Forum. 2026 Mar 20;7(3):e260034. doi: 10.1001/jamahealthforum.2026.0034 (PMC13005156; doi:10.1001/jamahealthforum.2026.0034)
Supplement: Supplement 2. — Data Sharing Statement [file jamahealthforum-e260034-s002.pdf]

Data Sharing Statement

Huguet. Insurance Churn and Diabetes Outcomes Among Patients With Low Income. *JAMA Health Forum*. Published March 20, 2026. doi:10.1001/jamahealthforum.2026.0034

Data
Data available: No

Additional Information
Explanation for why data not available: Raw data underlying this article were generated from multiple health systems across the ADVANCE network; restrictions apply to the availability and re-release of data under organizational agreements.
